# Metabolic Perturbations in a *Bacillus subtilis* *clpP* Mutant during Glucose Starvation

**DOI:** 10.3390/metabo7040063

**Published:** 2017-11-24

**Authors:** Daniel Schultz, Rabea Schlüter, Ulf Gerth, Michael Lalk

**Affiliations:** 1 Institute of Biochemistry, University of Greifswald, 17487 Greifswald, Germany; daniel.schultz@uni-greifswald.de; 2 Imaging Center of the Department of Biology, University of Greifswald, 17487 Greifswald, Germany; rabea.schlueter@uni-greifswald.de; 3 Institute of Microbiology, University of Greifswald, 17487 Greifswald, Germany; ulf.gerth@uni-greifswald.de

**Keywords:** Clp proteases, cell wall metabolism, metabolism under glucose starvation

## Abstract

Proteolysis is essential for all living organisms to maintain the protein homeostasis and to adapt to changing environmental conditions. ClpP is the main protease in *Bacillus subtilis*, and forms complexes with different Clp ATPases. These complexes play crucial roles during heat stress, but also in sporulation or cell morphology. Especially enzymes of cell wall-, amino acid-, and nucleic acid biosynthesis are known substrates of the protease ClpP during glucose starvation. The aim of this study was to analyze the influence of a *clpP* mutation on the metabolism in different growth phases and to search for putative new ClpP substrates. Therefore, *B. subtilis* 168 cells and an isogenic ∆*clpP* mutant were cultivated in a chemical defined medium, and the metabolome was analyzed by a combination of ^1^H-NMR, HPLC-MS, and GC-MS. Additionally, the cell morphology was investigated by electron microscopy. The *clpP* mutant showed higher levels of most glycolytic metabolites, the intermediates of the citric acid cycle, amino acids, and peptidoglycan precursors when compared to the wild-type. A strong secretion of overflow metabolites could be detected in the exo-metabolome of the *clpP* mutant. Furthermore, a massive increase was observed for the teichoic acid metabolite CDP-glycerol in combination with a swelling of the cell wall. Our results show a recognizable correlation between the metabolome and the corresponding proteome data of *B. subtilis* *clpP* mutant. Moreover, our results suggest an influence of ClpP on Tag proteins that are responsible for teichoic acids biosynthesis.

## 1. Introduction

*Bacillus subtilis* is a gram-positive soil bacterium with an intracellular protein degradation system consisting of different proteases [1,2]. Clp proteases seem to be the main proteolytic system in *B. subtilis* [3,4], and they are highly conserved in eubacteria except mycoplasma [5]. Furthermore, ClpP forms complexes with AAA+ ATPases (ATP that is associated with a variety of cellular activities). In *B. subtilis*, three different Clp ATPases with chaperone activity are known: ClpX, ClpC, and ClpE [6,7]. They form hexameric rings that interact with the protease ClpP and are responsible for substrate recognition and protein unfolding [8]. ClpP forms two heptameric ring complexes [9] and degrades selected proteins. These complexes are located at the polar and the mid-cell region together to their chaperones [10]. ClpP-mediated proteolysis is important for the degradation of denatured and aggregated proteins that can even be formed under standard conditions [11] or under stress conditions like heat stress [3,12]. Because of its general function in proteolysis, it is not striking that ClpP is involved in processes like sporulation [12], competence development [13], motility [14], biofilm formation [15], DNA repair [16], and virulence [17].

Under non-growing conditions, ClpP shuts down central metabolic pathways [6]. In *B. subtilis*, a couple of proteins are known ClpP substrates [6], such as the glutamine-fructose-6-phosphate transaminase (GlmS) and the UDP-N-acetylglucosamine 1-carboxyvinyltransferase (MurAA), the committed enzyme in the first step of cell wall biosynthesis [18]. Other ClpP substrates are part of amino acid biosynthesis, nucleotide synthesis, glycolysis, and vitamin metabolism [6]. For other gram-positive bacteria, like *Staphylococcus aureus*, similar results and even more ClpP substrates are known [19,20].

The aim of this study was to analyze the influence of a ClpP mutation on the citric acid cycle (TCA) and the cell wall metabolism in the different growth phases: exponential, transient, early, and late stationary phase. A combination of ^1^H-NMR (detection of extracellular metabolites and nutrition compounds), HPLC-MS (analysis of nucleotides, glycolytic and cell wall metabolites as well as cofactors), and GC-MS (measurement of amino acids and TCA metabolites) was used for the analysis of the metabolic fingerprint and footprint in order to correlate metabolic data with proteolysis of particular ClpP substrates.

## 2. Results

### 2.1. Extracellular Metabolites

**Figure 1:**
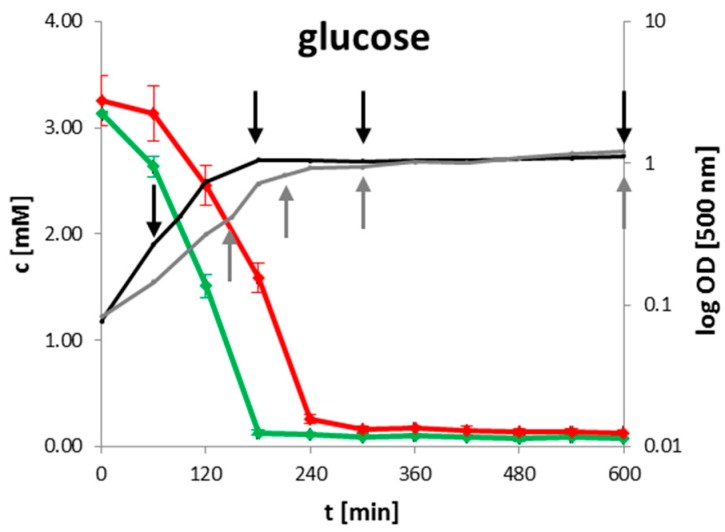

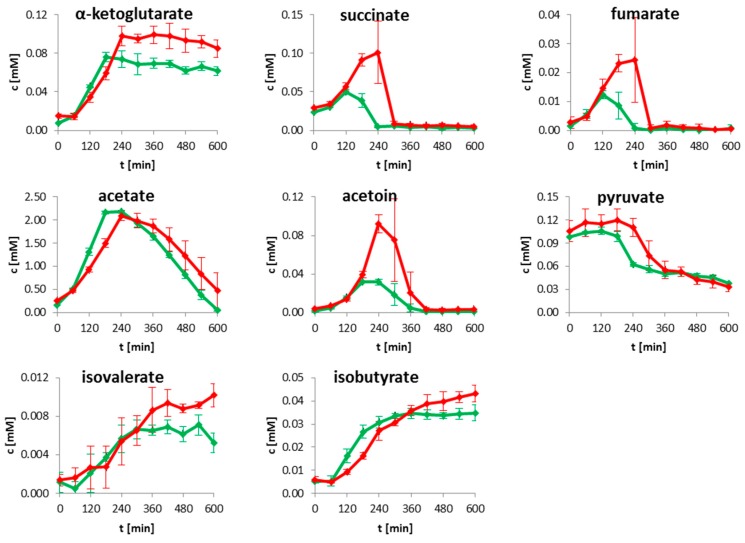
Time resolved quantification of selected metabolites for wild type (green) and *clpP* mutant (red). The growth curve for wild type (black) and mutant (grey) are illustrated together with the intracellular sampling time points (black and grey arrows) on the left side. Data are shown for four biological replicates.

For high throughput identification and quantification of uptake and secretion of overflow metabolites and nutrients during growth, ^1^H-NMR spectroscopy was used. Changes of metabolite concentrations of *B. subtilis* wild type and *clpP* mutant are displayed in Figure 1 as time series. Glucose as main carbon source was completely consumed when the cells entered transient growth phase after 180 min for the wild type and after 240 min for the mutant, respectively. The growth behavior of the mutant was retarded (Figure 1 and Supplementary Figure S1). An enhanced secretion of TCA metabolites succinate and fumarate and of the overflow metabolite acetoin especially during exponential growth was observed for the *clpP* mutant (Figure 1). *B. subtilis* secretes acetoin under anaerobic and aerobic conditions through the acetolactate synthase AlsS and acetolactate decarboxylase (AlsD) [21]. The secretion of acetoin has no effect on pH-value. Furthermore, the acetoin dehydrogenase (AcoABC) [22,23] can convert acetoin to 2,3 butanediol, which was not detected in the media of growing cells. Additionally, a slightly enhanced secretion of branched chain amino acid degradation intermediates like isobutyric acid and isovaleric acid was observed in the mutant when compared to the wild type during the stationary growth. After glucose consumption, the secreted metabolites were mostly consumed in both strains.

### 2.2. Intracellular Metabolites

#### 2.2.1. Glycolysis and Tricarboxylic Acid Cycle

**Figure 2:**
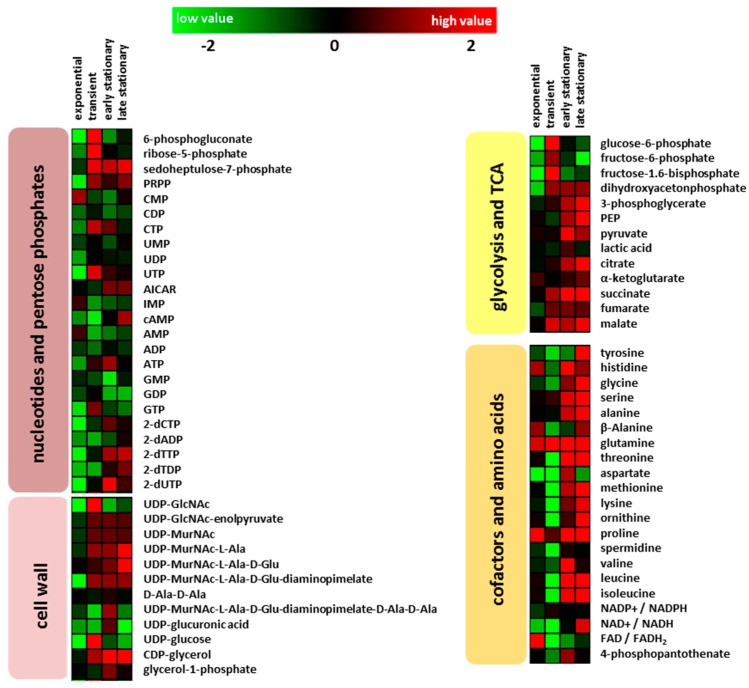
Color-coded heatmaps displaying fold change log_2_ (*clpP* mutant/wild type) of intracellular metabolite amounts for the different growth phases (exponential: for wild type after 90 min and mutant after 150 min; transient: for wild type after 180 min and mutant after 210 min; early stationary: for both after 300 min and late stationary growth phase: for both after 600 min) of four biological replicates.

76 intracellular metabolites were analyzed by GC-MS and HPLC-MS, and the retention times together with the mass spectra were compared with the respective from pure chemical standards. The glycolytic intermediates glucose-6-phosphate, fructose-6-phosphate, and fructose-1.6 bisphosphate were decreased during exponential growth in the *clpP* mutant (Figure 2). Under glucose starvation (absence of glucose in the media, see Figure 1), enhanced amounts of 3-phosphoglycerate and phosphoenolpyruvate were observed in the mutant. During exponential growth no significant differences (*p* > 0.05) in the amount of TCA metabolites citrate, 2-oxoglutarate, malate, and succinate could be detected in the wild type and the *clpP* mutant. The amount of fumarate was significantly (*p* < 0.05) decreased in the mutant. The metabolites succinate and malate were significantly (*p* < 0.05) increased in the transient growth phase (Figure 2). Moreover, for fumarate a slightly enhanced intracellular amount was observed during transient phase. In the exometabolome, a secretion of succinate and fumarate was found. The secretion of these metabolites is related to overflow in carbon metabolism due to high rates of respiration. In later growth phases, these compounds can serve as nutrients and they are therefore taken up (Figure 1). During glucose starvation an influence of the *clpP* deletion on TCA became noticeable. Except for oxoglutaric acid all of the other TCA intermediates were increased, whereas the amount of TCA metabolites in the wild type remains stable (Figure 2).

#### 2.2.2. Amino Acids and Nucleotides

**Figure 3:**
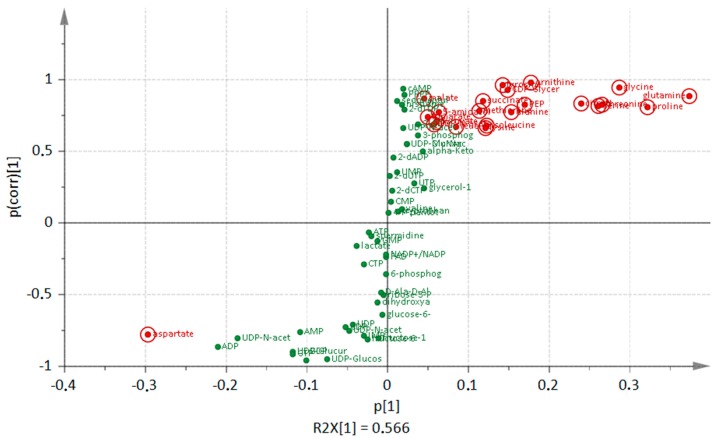
S-plot from Orthogonal Projections to Latent Structures Discriminant Analysis (OPLS-DA) loadings. The most increased and decreased metabolites (green dots) of the *clpP* mutant in comparison to the wild type in the late stationary phase are highlighted in red.

A deletion of *clpP* leads to many alterations in amino acid amounts of the cells that are dependent on growth phase (Figure 1). During transient growth phase, the intracellular levels of branched-chain amino acids, aspartate, lysine, threonine, tyrosine, and ornithine were significantly (*p* < 0.05) reduced in the mutant. In the stationary phase, especially in the late stationary growth phase, all of the detected amino acids except for aspartate were found in higher amounts in the mutant when compared to the wild type. Earlier experiments [6] showed that intracellular protein degradation mainly occured several hours after the cells entered the stationary growth phase (late stationary phase). Visualization of the Orthogonal Projections to Latent Structures Discriminant Analysis (OPLS-DA), as shown in Figure 3, illustrates how the amino acids dominate the group of intracellular metabolites. The S-plot can be used to identify unique and shared structures in the samples. This model shows that amino acids like proline, glutamine, and glycine with high p[1] and p_(corr)_ values are useful to charaterize the differences between both strains. Furthermore, the intermediate of inosine monophosphate, 5-aminoimidazole-4-carboxamide ribonucleotide (AICAR), and the second messenger cAMP were increased in the mutant reaching the stationary phase (Figure 2). Two forms of cAMP (namely 3′,5′-cAMP and 2,3′-cAMP) exists in bacteria [24], which cannot be distinguish at this stage with our analytical methods.

#### 2.2.3. Cell Wall Metabolism

**Figure 4:**
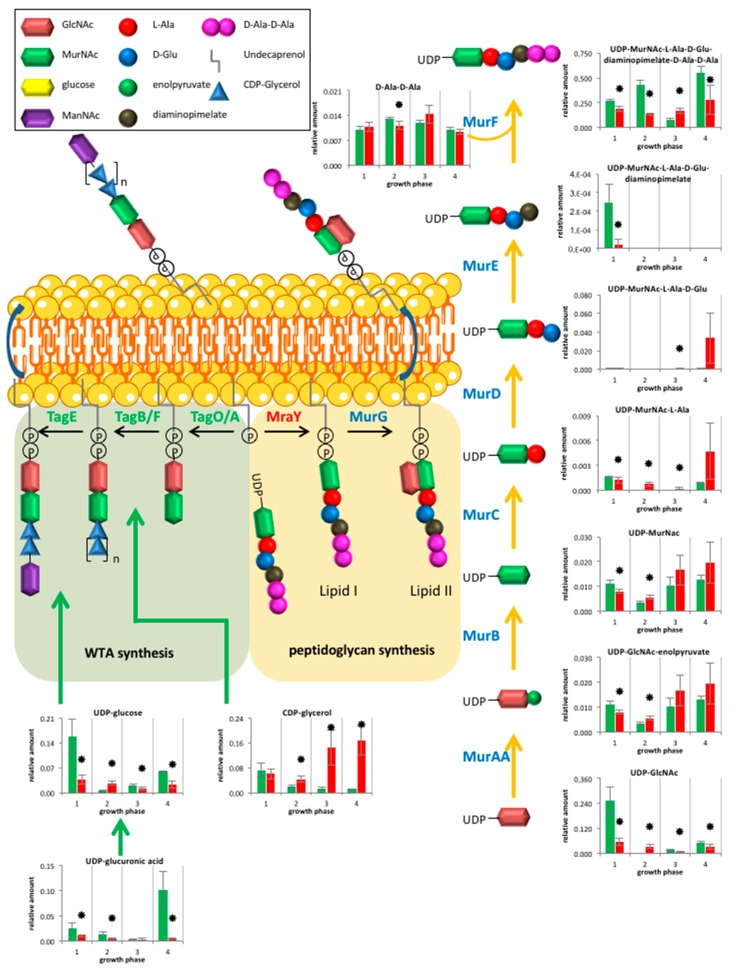
Overview of metabolites from peptidoglycan and Wall teichoic acids (WTA) synthesis for wild type (green) and *clpP* mutant (red) from the exponential growth phase (1), the transient (2), the early stationary (3) to late stationary growth (4). Data are shown for four biological replicates. Asterisks indicate significant changes (*p* value ≤ 0.05).

**Figure 5:**
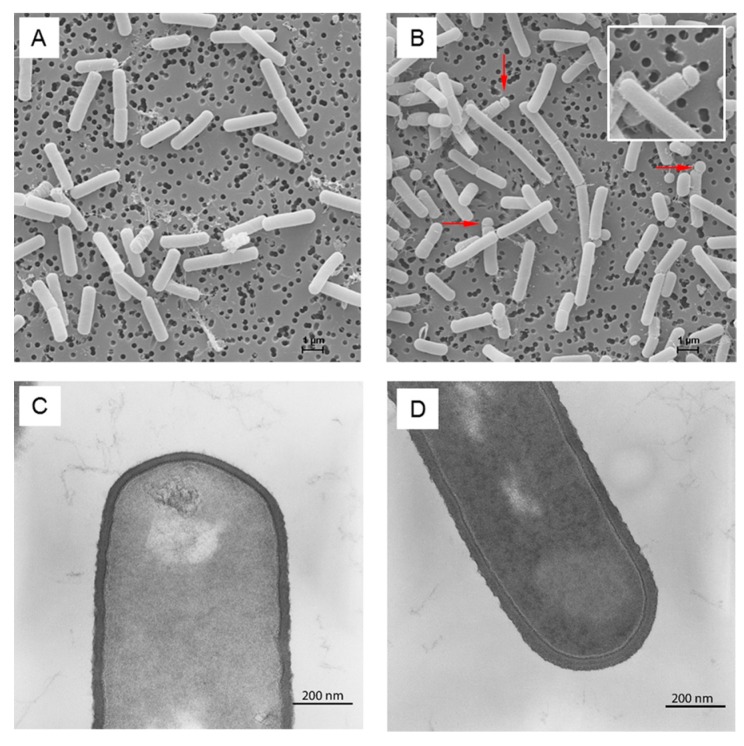
Scanning and transmission electron micrographs of *B. subtilis* wild type (**A**,**C**), respectively and *clpP* mutant (**B**,**D**), respectively during late stationary phase in minimal medium. Putative minicells and cell chains are marked by arrows (**B**).

The analysis of peptidoglycan and teichoic acid metabolites revealed a huge influence of *clpP* deletion. Enhanced amounts of the peptidoglycan intermediates UDP-GlcNAc-enolpyruvate and UDP-N-acetylmuramoyl-L-alanine were detected in the mutant from transient growth phase (Figure 4). Wall teichoic acids (WTA) of *B. subtilis* 168 consist mainly of polyglycerol phosphate. For their formation, the essential glycerol-3-phosphate cytidylyltransferase (TagD) uses CDP-glycerol as active precursor and building block [25,26]. We were able to detect increased amounts of CDP-glycerol under glucose starvation in the *clpP* mutant (Figure 4). We also noticed a significant swelling (*p* = 0.015) of the cell wall from 36 nm ± 2 nm to 60 nm ± 7nm during glucose starvation (Figure 5 and Supplementary Figure S2). Additionally, we detected decreased amounts of UDP-glucose in the mutant except for transient growth phase. 

The influence of antibiotics that targets peptidoglycan (fosfomycin, tunicamycin) or WTA synthesis (tunicamycin, ticlopidine) on both strains was also tested (Supplementary Figure S3). The *clpP* mutant exhibited a decreased resistance against fosfomycin and against the combinations of fosfomycin or tunicamycin with ticlopidine in comparison to the wild type. Only for tunicamycin alone, was an increased resistance for the mutant was observed. The reason for this phenomenon is so far not known; perhaps the *clpP* mutant is generally more susceptible for biologically active substances.

As shown by scanning electron microscopy, the cells of the mutant formed putative minicells and long cell chains [27] in minimal medium, probably due to cell separation disorders (Figure 5). 

## 3. Discussion

### 3.1. Comparison of Metabolic Alterations in Wild Type and clpP Mutant Cells

Through our metabolic analysis we were able to match 90% of known ClpP subtrates for *B. subtilis* based on former studies [6] with corresponding metabolites that are detectable by a combination of ^1^H-NMR and mass spectrometry based analysis. 

The majority of Clp targets are involved in amino acid biosynthesis and salvage pathways (for an overview see Supplementary Table S1)*.* The aromatic amino acids tryptophan and histidine are products of the ClpP substrate 3-deoxy-d-arabino-heptulosonate 7-phosphate synthase (AroA) [6]. The corresponding proteins for branched chain amino acid biosynthesis like threonine dehydratase (IlvA), acetolactate synthase (IlvB), and proteins responsible for leucine production (LeuA, LeuB, and LeuC) are additional ClpP substrates [6]. The proteins involved in methionine formation and salvage pathways methionine synthase namely (MetE), 5-methylthioribose kinase (MtnK), and isomerase (MtnS) are further ClpP targets [6], leading to a higher amount of methionine in the *clpP* mutant. An enhanced amount of lysine in the mutant is in agreement with another ClpP substrates: aspartokinase (LysC) [6]. The amount of other amino acids like glutamine, proline, glycine, and threonine were also increased in the *clpP* mutant under glucose starvation (Figure 3). Proline, as compatible solute, shows an accumulation under salt stress [28], and can suppress the formation of toxic protein aggregates [29], which occurred in *clpP* mutants [19]. Glutamine itself is the substrate for GlmS, a further known ClpP target [6]. The only amino acid that was found in decreased amounts was aspartate (Figure 3). Aspartate is a substrate for PyrB, which is involved in the synthesis of pyrimidine nucleotides and also targeted by ClpCP for proteolysis during glucose starvation [6]. A blocked proteolysis of PyrB seems to result in a higher turnover of aspartate.

The fact that under glucose starvation, proteins that involved in TCA are not affected by degradation is known for *S. aureus* cells [19]. This seems also be the case for *B. subtilis*, since citrate synthase CitZ and isocitrate dehydrogenase CitC are not targeted by ClpP-depend degradation (unpublished data) despite the elevated levels of TCA metabolites in the *clpP* mutant.

### 3.2. Cell Wall Metabolism and Morphology

The UDP-*N*-acetylglucosamine 1-carboxyvinyltransferase (MurAA) and the glutamine-fructose-6-phosphate transaminase (GlmS) are known ClpP substrates in *B. subtilis* [6], which leads to enhanced amounts of the corresponding metabolite UDP-GlcNAc-enolpyruvate (Figure 4). The UDP-*N*-acetylmuramoyl-l-alanine synthetase (MurC) is a known ClpP target in *S. aureus* [17] and catalyzes the formation of UDP-*N*-acetylmuramoyl-l-alanine, which was also found in increased amounts in the mutant entering glucose starvation. Thus, MurC may also be affected by ClpP dependent degradation in *B. subtilis*. However, not all of the detected metabolites from peptidoglycan biosynthesis showed an increase suggesting a complex regulation. 

The metabolite CDP-glycerol is essential for the formation of WTA. The massive increase of cell wall thickness (Figure 5 and Supplementary Figure S2) could be associated with the high amount of CDP-glycerol in the mutant, suggesting an enhanced WTA synthesis. WTA seem to be dispensable for cell viability in *B. subtilis* [30]. Whereas, in *B. anthracis* cells lacking TagO, the enzyme that initiates synthesis of murein linkage units, cannot maintain cell shape or support vegetative growth [31]. UDP-glucose is used for obligatory WTA glycosylation [32]. This metabolite plays also a role in cell division by regulation of UDP-glucose diacylglycerol glucosyltransferase (UtgP). A reduced amount of UDP-glucose can lead to a reduction of cell size [33], as we found for a subpopulation of mutant cells (Supplementary Figure S2).

ClpP seems to be involved in cell division and cell separation. This confirms, among other things, autolysins that are necessary for cell wall modifications [34]. For *S. aureus*, a daptomycin-resistant strain is known with a *clpP* mutation with a decreased autolysin production [35]. The possible formation of minicells is the result of asymmetric cell separation, which is known for *B. subtilis* since 1971 [36]. Proteins that are involved in cell division are part of the divisome. Key players in this process are the Fts and Min proteins. An absence or overexpression of these proteins results in minicell formation, possibly through additional cell division events per cell cycle when FtsZ is overexpressed [37]. For *S. aureus* cell-division initiation protein (FtsZ) and the cell division protein (FtsA) are known ClpP substrates [38], and FtsZ is also a ClpP target for *B. subtilis* under treatment with acyldepsipeptides [39].

## 4. Materials and Methods

### 4.1. Cultivation

*Bacillus subtilis* 168 and an isogenic *clpP* mutant [6] were grown in modified M9 medium [40] containing 2.7 mM glucose, 4.5 mM glutamic acid, 0.8 mM tryptophan, 0.01% yeast extract to allow for the growth of the *clpP* mutant, 0.5 mM MgSO_4_, 0.001 mM FeSO_4_, and 0.01 mM MnSO_4_. The main culture was inoculated with an overnight culture in exponential growth phase (optical density at 500 nm between 0.4 and 0.7) at an optical density of 0.08. The overnight culture of the mutant contained a final concentration of 200 µg/mL spectinomycin sulfate. Cultivation was carried out in 1000 mL shake flasks with 200 mL medium at 37 °C and 180 rpm on a rotary shaker. 

### 4.2. Sampling of Extracellular Metabolites

During cultivation, every 60 min 2 mL of cell culture was rapidly sterile syringe filtered (0.45-µm-pore-size, Sarstedt AG, Sevelen, Switzerland) according to the protocol of Meyer et al. [41]. Samples were stored at −20 °C before measurement.

### 4.3. ^1^H-NMR Spectroscopic Analysis of Extracellular Metabolites

400 µL of sample was mixed with 200 µL 0.2 M sodium hydrogen phosphate butter (pH = 7.0, containing 1 mM 3-(trimethylsilyl)propionic-2,2,3,3-d_4_ acid sodium salt) made up with 50% D_2_O to provide a nuclear magnetic resonance (NMR)-lock signal. NMR spectra were obtained at 600.27 MHz at a temperature of 310 K, using a Bruker^®^ Avance-II 600 NMR spectrometer operated by TOPSPIN 3.2 software (Bruker Biospin GmbH, Fällanden, Switzerland). Spectral referencing was done relative to the TSP signal. Data analysis (identification and quantification) was done using AMIX v3.9.14 software (Bruker Biospin GmbH, Fällanden, Switzerland). The detection limit was usually in the µmol range. For further description see Dörries et al. [42].

### 4.4. Sampling of Intracellular Metabolites

For analysis of the intracellular metabolites samples were taken at four different growth phases of the cells: exponential phase (wild type 90 min and mutant 150 min after inoculation), transient phase (wild type 180 min and mutant 210 min after inoculation), early stationary phase (for both after 300 min), and late stationary phase (for both after 600 min). Samples were obtained by a fast vacuum dependent filtration, according to the method of Meyer et al. [41]. For this, 20 OD units of the cell culture were separated from the medium by fast filtration and washed twice with 20 mL ice-cold isotonic NaCl solution. The filter with the bacterial cells was immediately transferred into a falcon tube that was filled with 5 mL ice-cold extraction solution (60% ethanol, *w*/*v*). All of the samples were quickly frozen with liquid nitrogen and stored at −80 °C. The following steps were done on ice. For metabolite extraction and cell disruption the cells were thawed and internal standards were added (for GC-MS: 20 nmol ribitol, norvaline, *N*,*N*-dimethyl-phenylalanine, and *p*-chloro-phenylalanine-hydroxide, and for LC-MS: 2.5 nmol camphorsulfonic acid). Subsequently, samples were 10 times alternately shaken and vortexed. After centrifugation for 5 min at 4 °C and 10,015 *g*, the supernatant was transferred into a new tube and a second extraction was done using ice-cold water. Both of the supernatants were merged, diluted with 20 mL ice-cold water, and stored at −80 °C before lyophilization.

### 4.5. GC-MS Analysis of Intracellular Metabolites

Lyophilized samples were derivatized for 90 min at 37 °C with 40 µL methoxyamine (20 mg/mL pyridine) and then for 30 min at 37 °C with 80 µL *N*-methyl-*N-*trimethylsilyltrifluoroacetamide. After centrifugation for 3 min at room temperature, the supernatant was transferred into GC-Vials for measurement. Analyses were performed by using a GC-MS (EI, quadrupole) and a DB-5MS column (30 m × 0.25mm × 0.25 µm). Parameters of GC-MS and oven program were described in Dörries et al. [43]. The identification of metabolites was done by comparison of retention time and fragmentation patterns of detected peaks and those of standard compounds measured with the same method. For a list of all the identified metabolites and their amounts see in supplementary information. The area of the quantifier ion was integrated and normalized to the quantifier ion of internal standard *N*,*N*-Dimethyl-l-phenylalanine by using the Chroma^®^TOF^®^ V4.50.8.0 (Saint Joseph, MI, USA). Detection limit was usually in the nmol range.

### 4.6. HPLC-MC Analysis of Intracellular Metabolites

Lyophilized samples were dissolved in 100 µL water and centrifuged at room temperature for 3 min. The supernatant was transferred into a glass vial with micro insert. Ion-pairing-LC-MS measurement was performed by using an Agilent HPLC system, consisting of a degasser, a quaternary pump, and an autosampler [41]. The system was coupled to a Bruker micrOTOF mass spectrometer (Billerica, MA, USA). The chromatic separation was performed using a RP-C_18_ column (3.5 mm × 150 mm × 4.6 mm) with a C_18_ pre-column. The mobile phase composition was: (A) 5% methanol and 95% water, containing 10 mM tributylamine as ion-pairing reagent, 15 mM acetic acid for pH adjustment to pH 4.9 and (B) 100% methanol, as previously described [44]. The mass spectrometer operating in ESI negative mode was used over a mass range from 50 to 3000 *m*/*z*. Metabolite identification was carried out by a comparison of retention time and mass spectra with an in-house database and HMDB [45]. Quantification of metabolite signals was done by QuantAnalysis 2.0. For normalization, the internal standard camphor sulfonic acid was used. Detection limit was usually in the nmol range. The energy charge (EC) was calculated with absolute concentrations of ATP, ADP and AMP. For this purpose calibration curves were used. The curve fitting was done by a 1/x weighting using a quadratic calibration mode. The following equation [46] was used: EC = ([ATP] + 0.5 [ADP])/([ATP] + [ADP] + [AMP]).

### 4.7. Statistical Analysis and Visualization

Significance tests for the mean value of four biological replicates were performed using an unpaired *t* test from VANTED [47] V2.2.1 with *p*-values of 0.05. For visualization of the metabolome data Microsoft Excel^®^2007 was used. Heatmaps were created by MeV [48] V4.9.0 and OPLS-DA were done by SIMCA 14.1. For this purpose, the pareto scaling was used.

### 4.8. Transmission Electron Microscopy

The cells were fixed (1% glutaraldehyde, 4% paraformaldehyde, 50 mM sodium azide in 5 mM HEPES buffer pH 7.4) at 4 °C, and stored at the same temperature until further processing. After that, cells were treated with 0.5% glutaraldehyde and 1% osmium tetroxide in washing buffer (100 mM cacodylate buffer [pH 7.0], 1 mM calcium chloride, 0.09 M sucrose) for 1 h at 4 °C. Subsequent to embedding in low gelling agarose, cells were postfixed in 2% osmium tetroxide in washing buffer for 1 h at room temperature, and then stained with 0.5% uranyl acetate in 0.9% sodium chloride at 4 °C overnight. After dehydration in graded series of ethanol (30–100% ethanol), the material was transferred stepwise into propylene oxide and was finally embedded in AGAR-LV resin (Plano, Wetzlar, Germany). Sections were cut on an ultramicrotome (Reichert Ultracut, Leica UK Ltd., Milton Keynes, UK), stained with 4% aqueous uranyl acetate for 5 min followed by lead citrate for 1 min, and analysed with a transmission electron microscope LEO 906 (Carl Zeiss Microscopy GmbH, Oberkochen, Germany).

### 4.9. Scanning Electron Microscopy

In the investigated growth phases, the samples of eight OD units of bacterial culture were obtained by vacuum depend filtration on a 0.2 µm polycarbonate filter (Merck, KGaA, Gernsheim, Germany). A part of 1 cm^2^ was transferred into fixation solution. After a fixation step (1% glutaraldehyde, 4% paraformaldehyde, 50 mM sodium azide in 5 mM HEPES [pH 7.5]), samples were treated with 2% tannic acid in washing buffer (100 mM cacodylate buffer [pH 7.0], 1 mM calcium chloride, 50 mM sodium azide) for 1 h, 1% osmium tetroxide in washing buffer for 1 h, and 1% thiocarbohydrazide for 30 min at room temperature—with washing steps in between. After treatment with 1% osmium tetroxide in washing buffer over night at 4 °C, the samples were dehydrated in a graded series of aqueous ethanol solutions (10%, 30%, 50%, 70%, 90%, 100%) on ice for 15 min each step. Before the final change of 100% ethanol, the samples were allowed to reach room temperature and then critical point-dried with liquid CO_2_. Finally, samples were mounted on aluminum stubs, sputtered with gold/palladium and examined with a scanning electron microscope EVO LS10 (Carl Zeiss Microscopy GmbH, Oberkochen, Germany).

### 4.10. Disk Diffusion Experiments

Cells were cultivated in modified M9 media described previously until the cells reached the exponential growth phase. 250 µL of culture were spread onto M9 agar plates. A filter disk (6 mm diameter) with antibiotic (20 µL per disk) was placed on top of the plates. We performed antibiotic sensitivity test with fosfomycin (target: UDP-*N*-acetylglucosamine 1-carboxyvinyltransferase MurA), tunicamycin (inhibits glycosylation of peptidoglycan and wall teichoic acids) and ticlopidine (WTA inhibitor) using 2 times minimal inhibitory concentration for antibiotics alone and 1 time MIC for a combination of fosfomycin/tunicamycin with ticlopidine [49,50,51,52]. The following concentrations were used: fosfomycin (1600 µg/mL), tunicamycin (1.0 µg/mL), ticlopidine (256 µg/mL), and as control, kanamycin (8 µg/mL) and water. The plates were incubated at 37 °C for 24 h and the diameter of the blocking zone was measured. Experiments were performed in triplicates for wild type and mutant.

## 5. Conclusions

This study shows a broad influence of a *clpP* deletion on the exo- and endometabolom of *B. subtilis* under glucose starvation. Differences in the amounts of metabolites were mainly observed when the cells reached the stationary phase. The *clpP* mutant showed higher levels of most glycolytic metabolites, the TCA intermediates, as well as amino acids and peptidoglycan precursors when compared to the wild-type. During glucose starvation, wild-type cells shut down the central carbon metabolism by degrading the corresponding enzymes. The deletion of *clpP* mainly affects the proteolysis of selected enzymes of the citric acid cycle, amino acid, and cell wall biosynthesis. These results are in accordance with known ClpP substrates that emerged from proteome analyses of *B. subtilis* [6] and *S. aureus* [19]. Additionally, our metabolic approach delivered a hint for new putative ClpP substrates. This includes proteins like AlsS, AlsD, SucCD, and especially TagD. 

## Supplementary Materials

The following are available online at www.mdpi.com/2218-1989/7/4/63/s1, Figure S1: Growth curves of *B. subtilis* wild type (green solid line) and *clpP* mutant (red solid line) together with energy charge (dashed lines) of four biological replicates, Figure S2: Overview about cell length, Figure S3: Disk diffusion experiments using disks with 6 mm diameter, Table S1: List of detected and altered metabolites with their corresponding proteins known as ClpP substrates in the *clpP* mutant in comparison to the wild type.
