# Supplementary material for: Metabolic Perturbations in a Bacillus subtilis clpP Mutant during Glucose Starvation
Source: Metabolites. 2017 Nov 24;7(4):63. doi: 10.3390/metabo7040063 (PMC5746743; doi:10.3390/metabo7040063)
Supplement: Supplementary file 1 [file metabolites-07-00063-s001.zip › Supplemental_Material.docx]

**Supplemental Material**

**
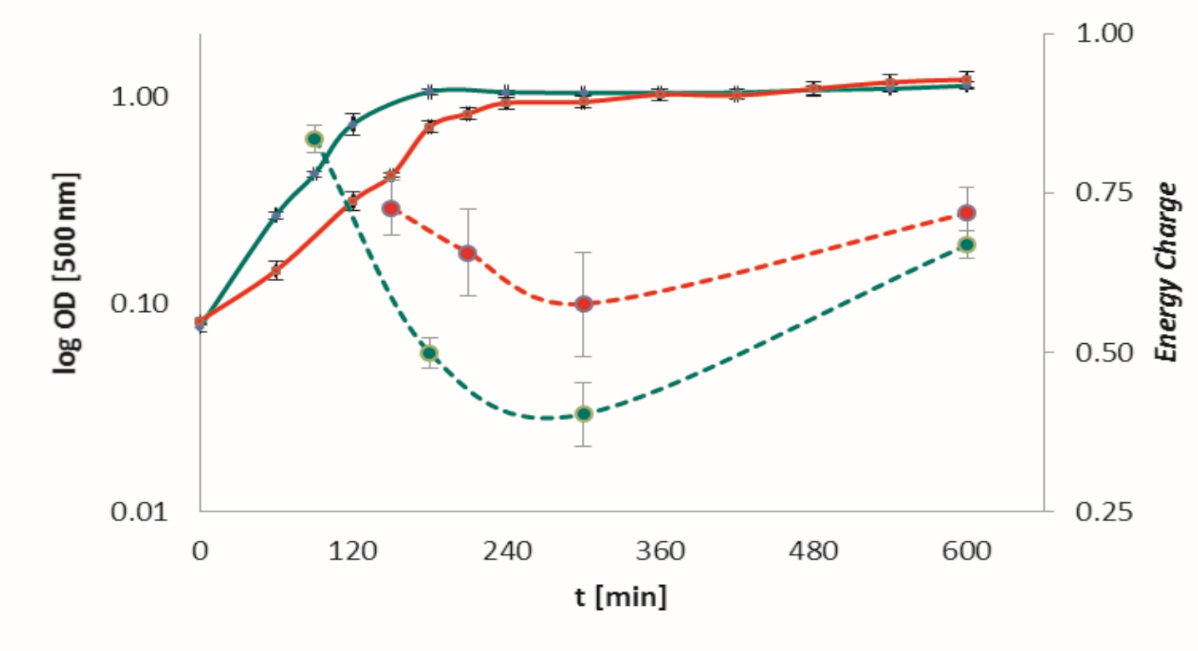
**

**Suppl. Figure 1:** Growth curves of B. subtilis wild type (green solid line) and clpP mutant (red solid line) together with energy charge (dashed lines) of four biological replicates.

**
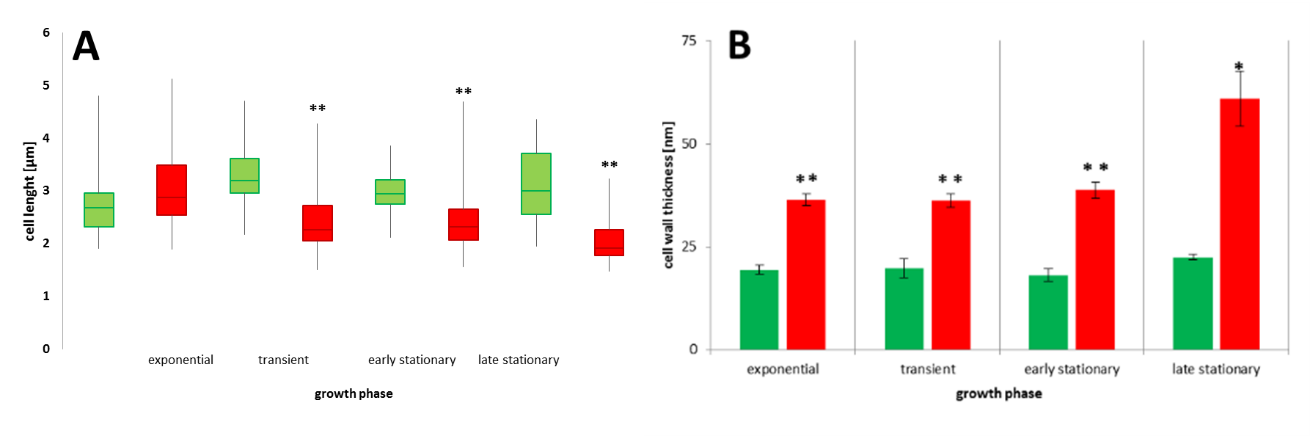
**

**Suppl. Figure 2:** Overview about cell length (A) illustrated as box plot and cell wall thickness (B) for B. subtilis wild type (green) and clpP mutant (red) during different growth phases in minimal medium. The cell length was sized for 50 cells per strain and time point. The cell wall thickness was calculated by the average of three representative cell walls per strain and time point (* p < 0.05, ** p < 0.01).

**Suppl. Table 1**. List of detected and altered metabolites with their corresponding proteins known as ClpP substrates in the clpP mutant in comparison to the wild type.

| **metabolites** | **corresponding proteins** | **pathway** |
| --- | --- | --- |
| tyrosine | AroA1 | amino acid biosynthesis |
| isoleucine, valine | IlvB | amino acid biosynthesis |
| leucine | LeuA1, LeuA2, LeuC1, LeuC2, LeuD | amino acid biosynthesis |
| lysine | LsyC | amino acid biosynthesis |
| methionine | MetE, MtnK, MtnS | amino acid biosynthesis and salvage |
| AICAR | PurB | purine metabolism |
| aspartate | PyrB | pyrimidine metabolism |
| UDP-GlcNAc-enolpyruvate | MurAA | cell wall synthesis |
| UDP-MurNAc-L-Ala | MurC* | cell wall synthesis |

*known ClpP substrate for *S. aureus*.

**Suppl. Figure *3*:** Disk diffusion experiments using disks with 6 mm diameter. Growth-inhibitory zones are shown for wild type (green) and *clpP* mutant (red) for three replicates.
