# Supplementary figures and images for: Metabolic Perturbations in a Bacillus subtilis clpP Mutant during Glucose Starvation
Source: Metabolites. 2017 Nov 24;7(4):63. doi: 10.3390/metabo7040063 (PMC5746743; doi:10.3390/metabo7040063)

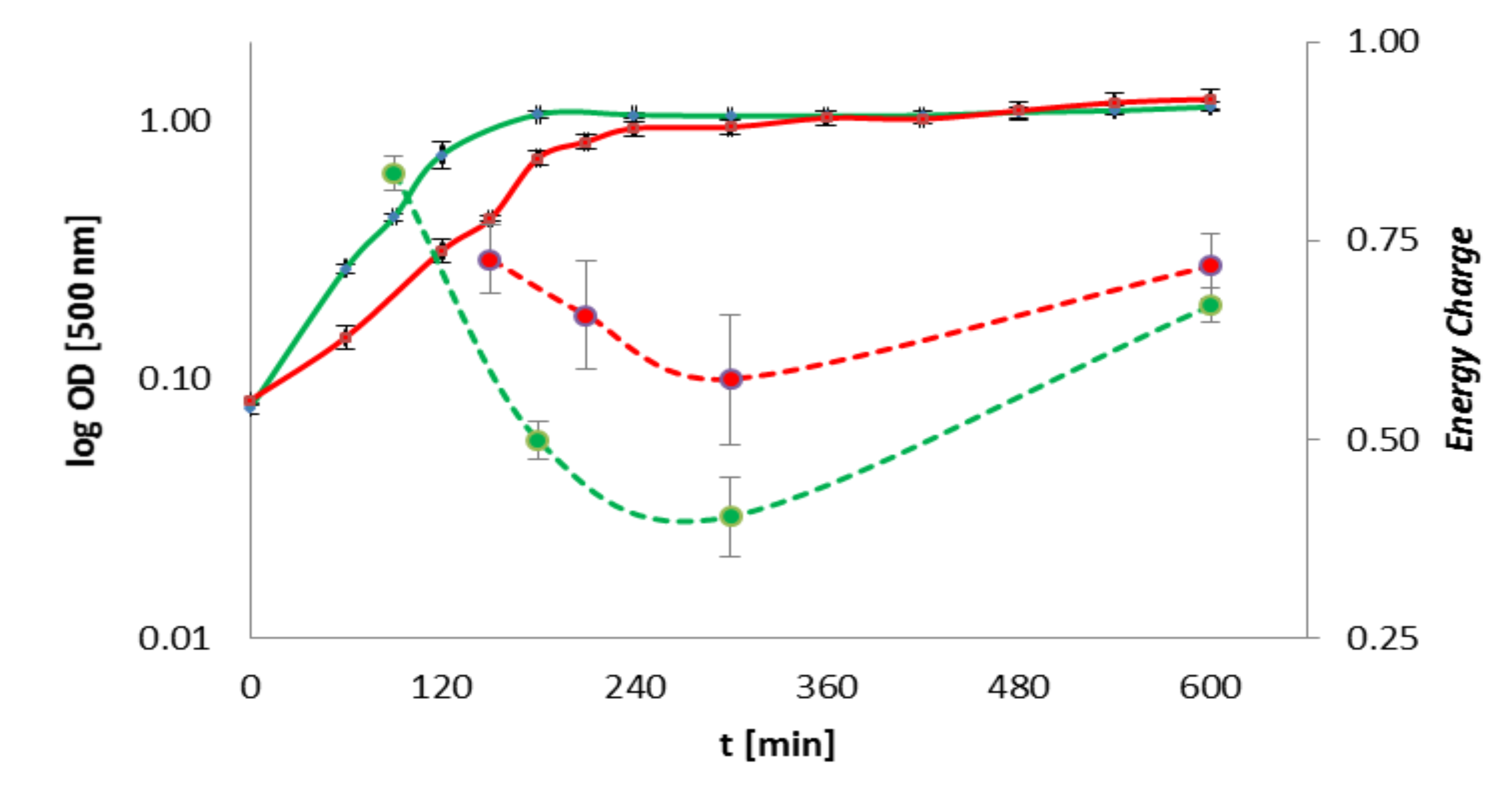

Supplement: Supplementary file 1 [file metabolites-07-00063-s001.zip › Suppl. Fig 1.tiff]

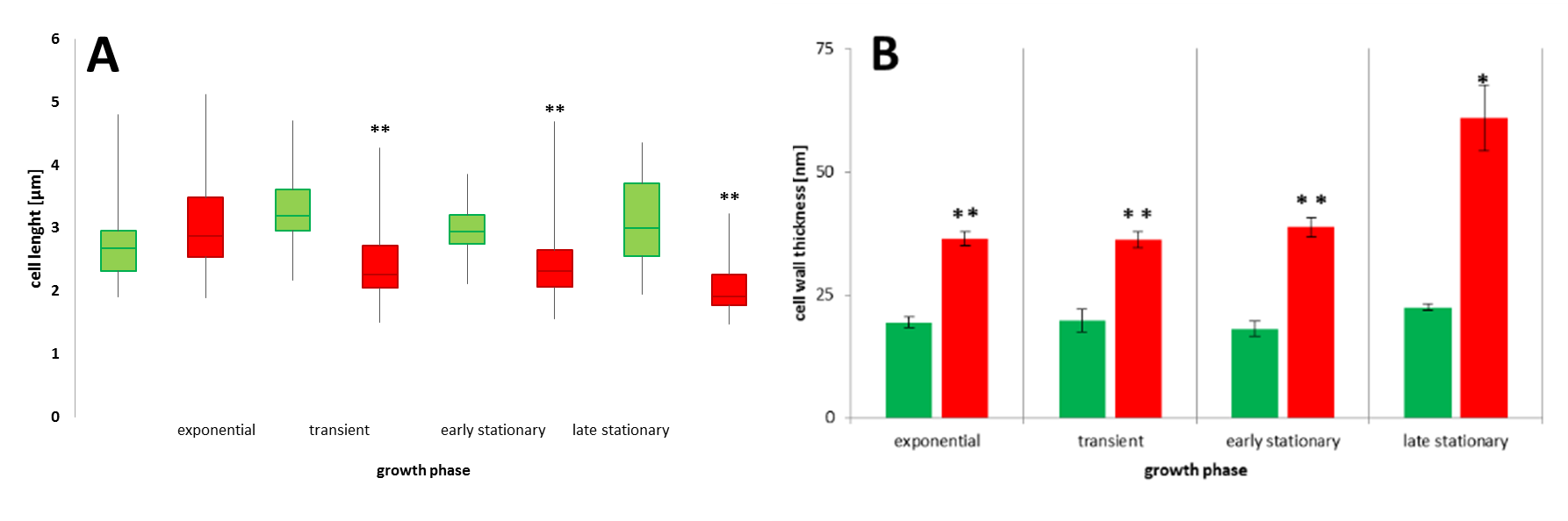

Supplement: Supplementary file 1 [file metabolites-07-00063-s001.zip › Suppl. Fig 2.tiff]

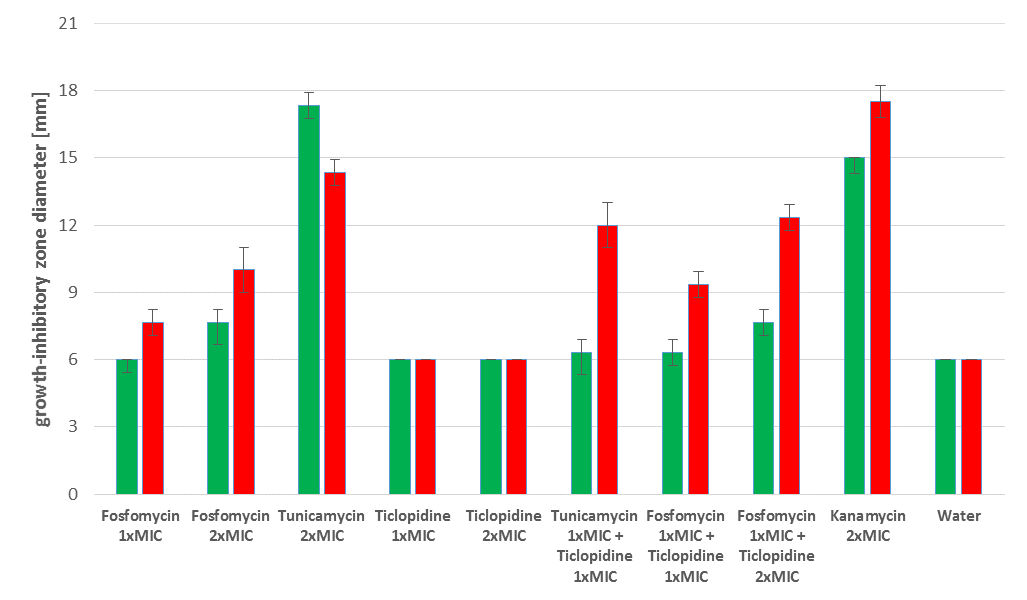

Supplement: Supplementary file 1 [file metabolites-07-00063-s001.zip › Suppl. Fig 3.tiff]
